# Integrated Analgesic Care in the Current Human Monkeypox Outbreak: Perspectives on an Integrated and Holistic Approach Combining Old Allies with Innovative Technologies

**DOI:** 10.3390/medicina58101454

**Published:** 2022-10-15

**Authors:** Guy Henri Hans, Davina Wildemeersch, Ine Meeus

**Affiliations:** 1 Antwerp University Hospital (UZA), Multidisciplinary Pain Center (PCT), Drie Eikenstraat 655, 2650 Edegem, Belgium; 2 Faculty of Medicine and Health Sciences, University of Antwerp (UA), 2610 Antwerp, Belgium

**Keywords:** monkeypox, human, pain, neuropathic pain, analgesia, symptom management, holistic care, digital health

## Abstract

Monkeypox (MPX) is a zoonotic infection caused by an orthopoxvirus that is endemic to Central and Western Africa. The MPX virus is a part of the same family of viruses as the variola virus, which causes smallpox. Since May 2022, there has been a global increase in the incidence of MPX infections in multiple countries where the illness is not usually prevalent. A growing number of publications have emphasized on the need for increased awareness among all health professionals for the rapid recognition and diagnosis of this disease and for proper public health measures. However, atypical presentations and occurrence of uncommon symptoms receive less than the desired attention. More specifically, MPX infection related nociceptive symptoms are currently underexposed. Nevertheless, reports from the current outbreak have revealed that (severe) pain is one of the major causes for distress and even hospitalization in these patients. As for all serious pain conditions, an integrated, multidisciplinary, and holistic approach is indicated. This approach should be multimodal and include non-pharmacological therapies alongside pharmacological approaches. Health care professionals should be aware of available alternatives when first choice analgesic therapies fail. Protocols for identification of pain type and prolonged monitoring of clinical status should be implemented to improve patient well-being during acute infection, but also prevent chronic nociceptive syndromes.

## 1. Introduction

Monkeypox (MPX), an orthopoxvirus, is a genus that includes the smallpox virus. This zoonotic agent was discovered in 1958 in Denmark among laboratory monkeys [1]. Following the first confirmed case of human monkeypox (MPX) in 1970, MPX was sporadically reported in small and large outbreaks in endemic countries in Africa [1,2,3]. Since early May 2022, cases of MPX have been reported from countries where the disease is not endemic, and continue to be reported in several endemic countries. This is the first time that many MPX cases and clusters have been reported concurrently in non-endemic and endemic countries. The outbreak has been spreading around the world rapidly. The unexpected appearance of MPX in several regions in the initial absence of epidemiological links to areas in West and Central Africa, suggests that there may have been undetected transmission for some time. By mid-September 2022, the World Health Organization (WHO) reported 64,561 laboratory confirmed cases in 105 countries across all 6 WHO regions, including 26 deaths. In addition, 3154 probable cases had been identified. On 23 July 2022, the WHO Director-General issued a statement that the global MPX outbreak represents a public health emergency of international concern [4].

MPX is commonly associated with a widespread chickenpox-like rash that evolves into fluid-filled blisters that eventually scab. Textbook description of MPX describes two distinct phases: a beginning with flu-like symptoms including fever, tiredness, and a single swollen lymph node, and a week later, rash appears, first on the face and then spreading over the extremities. Eventually, the rash may cover the trunk or even the whole body. 

Reports from the current outbreak suggest that clinical manifestations may however not follow the classic presentation of MPX [5,6,7]. Prodrome or systemic symptoms do not always occur or precede the rash. In addition, the rash seems to be much more limited compared with that in the classical presentation, often limited to only one or two small lesions [8]. Mucosal involvement occurs in approximately 40% of cases and includes genital, perianal, and oropharyngeal lesions. However, even if only a small part of the body is affected, MPX is not necessarily a mild illness. Indeed, an increasing number of patients have reported severe pain symptoms over a prolonged period. Recent reports indicate that severe pain is one of the main reasons for hospitalization. The hospitalization rate in Milan, Italy, was observed to be 8.8% [9]. Bacterial surinfection, proctitis and severe perianal pain were in this population the main reasons for hospitalization. A recent meta-analysis looking into the complications, hospitalizations and deaths associated with MPX reported a mean hospitalization rate of 35% (14–59%) [10]. A range of painful clinical manifestations were hereby described, ranging from minor nociceptive symptoms such as sore throat and headache, to more severe or even generalized nociceptive syndromes such as myalgias and arthralgias. 

Nociceptive symptoms should not be overlooked because they carry a risk for sustained nociceptive syndromes, as seen during the recent COVID-19 pandemic. Moreover, debilitating pain can induce severe mental distress and depression. Analgesic protocols need to be developed since they are mainly missing in current clinical management guidelines, both at a national Belgian level as well as in international guidelines such as WHO and CDC. First, observation methods to monitor patients’ global health during isolation period need to be urgently established. Second, a follow-up system to closely monitor the eventual development of long-lasting nociceptive syndromes, linked to the acute viral infection, is necessary.

## 2. Clinical Nociceptive Manifestations during the Current Outbreak

### 2.1. Rash

The rash, even when limited to one body region such as the genital and with only one or two poxes (as often observed in the current multi-country outbreak), can be very painful. It is currently somewhat typical that rash, lesions, or sores mainly occurred in areas that are hard to see, such as the genitals, anus, and anal area or on the face, arms, and legs. Skin lesions can be even present on the scalp. Deep ulcerations and scarring of skin lesions can induce pain, often with some neuropathic components due to destruction of cutaneous nociceptors.

### 2.2. Genital Manifestations

During the recent outbreak, some particular symptoms leading to pronounced pain in the infected patients have been described [7,11,12]. Penile swelling and rectal pain are two such symptoms reported by some patients with MPX in the recent outbreak, symptoms that are not generally associated with MPX. For example, a recent study described that in a group of 197 infected patients, 71 reported rectal pain and 31 reported penile swelling [11]. Some symptoms were so pronounced that, overall, 20 patients had to be hospitalized for symptom management, of which eight admissions were for anal or rectal pain and five were for penile swelling. In another study on infections across 16 countries describing 528 cases, 13% (*n* = 70) of the patients required hospitalization [5], of whom 21 were for pain management (primarily for severe anorectal pain). In a Spanish observational cohort study 181 patients with confirmed MPX diagnosis were included [12]. 78% of participants developed lesions in the anogenital region, and 43% in the oral and perioral region. 70 (39%) participants had complications requiring treatment, 45 patients had a proctitis, 19 had tonsillitis, 15 had painful penile oedema, 6 had an abscess, and 8 had an exanthem. 

### 2.3. Lymphadenopathies

Furthermore, a substantial number of patients present with swollen tonsils, another atypical symptom of MPX. The presence of swollen lymph nodes also induces significant pain in many patients, as recently shown in a German database [13]. Lymphadenopathy is most frequently observed in the inguinal region, followed by the cervical region. Approximately 16.8% of patients in the German registry reported sore throat and difficulty in swallowing because of cervical lymphadenopathy. Inguinal lymphadenopathy on the other hand often leads to groin pain but can also induce meralgia paresthetica or even entrapment of the femoral nerve with severe pain and sensory disturbances (such as tingling) in the leg [14,15,16].

### 2.4. A proposed Multidisciplinary Integrated Care Approach

There is very limited information on the analgesic approach of MPX-related infections in the current outbreak. A recent publication by Pfäfflin and colleagues provides some information on the pain symptomatology and management in six patients who were hospitalized in Berlin [17]. In this case, 5 out of 6 patients rated their pain as 9–10/10 on the numerical rating scale and described their stabbing and burning pain as unprecedented in severity. All patients were treated with a combination of systemic (sometimes intravenously) and local analgesics. Although severe, the pain described by these patients responded to analgesics. A challenging question that remains unanswered is whether administration of tecovirimat could alleviate such intense pain. 

Clinical care for MPX should be optimized to alleviate symptoms, manage complications, and prevent long-term sequelae. Considering the clinical manifestations observed during the current worldwide outbreak, specific and multimodal analgesic protocols should be developed and disseminated. A combination of topical treatment options and systemic treatment is often indicated. In addition, patients should be closely monitored throughout their clinical course to promptly detect the occurrence of long-lasting post-viral disturbances. Interestingly, some studies previously warned of both acute and prolonged nociceptive complications after MPX infection. For example, Adler et al. described the clinical features and management of human MPX on patients in the UK who had been diagnosed with MPX between 2018 and 2021 (before the current outbreak) [18]. Seven patients were included in this retrospective observational review. The authors described severe neuropathic pain in some of these patients requiring opioid analgesia. Therefore, we propose the implementation of a protocol for the supportive management of nociceptive symptoms in human MPX infections. Based on the available clinical and scientific data, we put together a comprehensive table as a guide for the supportive management of symptoms (Table 1). This approach should be multimodal and include non-pharmacological therapies alongside pharmacological approaches. Additional supportive care which are essential and valuable treatment options for MPX cases include rehydration, restoring the hemodynamic balance, providing supplemental oxygen, managing bacterial surinfections of skin lesions, and eye infections/complications by applying lubricants, topical antibiotics and topical antivirals such as trifluridine [19]. CDC and WHO have also published guidelines to manage and contain the current MPX outbreak, but these reports are rather limited in terms of provided analgesic treatment options [20,21]. A stepwise approach, based on the patients’ somatosensory complaints, is needed including the use of atypical analgesic drugs for neuropathic pain conditions. Pain and other symptoms should be addressed through a multimodal approach [22,23,24]. However, such approaches are now largely lacking in available national and international guidelines, which is why the summary table was drawn up (see Table 1).

Patients and clinicians should be aware that pain in many cases can be alleviated through the application of home remedies, including topical application of soothing substances, and application of non-invasive neuromodulation techniques such as transcutaneous nerve stimulation, transcranial Direct Current Stimulation (tDCS), or vagus nerve stimulation (VNS), which may have added value in the control of inflammation in addition to pain alleviation [26]. Considering the reported occurrence of neuropathic pain conditions during acute MPX infection, one should consider including a screening tool for neuropathic pain into the monitoring set of patients. Although screening tools are certainly not perfect, their biggest benefit is to assist the attending physician in the identification of neuropathic pain or pain that has a predominantly neuropathic component. In this regard, tools such as the Leeds Assessment of Neuropathic Symptoms and Signs (LANSS) and the Douleur Neuropathic and the 4 Questions (DN4) are preferred as they both use semi-objective simple bedside tests for abnormal neural function. 

Paracetamol is often described as the first-line treatment for fever and pain in human MPX infections. However, its use in clinical practice is probably tempered by lack of knowledge in healthcare workers and their limited confidence in the diagnosis and management of human monkeypox [27,28,29]. Evidence on the immunomodulatory effect of paracetamol in infectious diseases is limited and somewhat conflicting. In one study on mice, paracetamol attenuated influenza-induced immunopathology without compromising viral clearance morbidity [30]. In the same study, paracetamol reduced morbidity in vivo. In a human study however, paracetamol suppressed serum neutralizing antibody response in common cold [31]. In influenza, paracetamol had no effect on viral shedding or clinical symptoms overall [32]. Despite these results, paracetamol should still be considered as the first-line agent for fever and pain. If analgesic efficacy is insufficient, then paracetamol should be combined with an analgesic drug of higher potency.

In a 2019 challenge study on analgesia in prairie dogs with MPX [33], the investigators identified buprenorphine (BUP) as a promising analgesic drug for MPX-related pain symptoms. On the other hand, meloxicam was associated with higher morbidity and mortality trends compared with the control group or the BUP-treated animals. BUP is indeed an interesting candidate to serve as a second-line analgesic drug in case of persisting pain symptoms. If first-line treatment with paracetamol fails, BUP can be used as a second-line analgesic drug. BUP has shown efficacy in diverse nociceptive syndromes, including neuropathic pain conditions. Finally, it is interesting to mention that BUP induces viral suppression [34]. This could be of additional preventive value in MPX infections. BUP also shows protective effects against the development of myonecrotic disease specific to *Clostridium perfringens*-mediated myonecrosis [35]. If therapy with BUP fails for some reason or is not possible, treatment with another atypical opioid such as tapentadol may be initiated [36]. Tapentadol has a lower risk of opioid-related side effects, such as constipation and nausea, which is of high clinical importance in the MPX patient population. This analgesic drug is preferable in clinical conditions requiring immediate dose adjustments or in patients at high risk of adverse events [37]. In case of unavailability of tapentadol, tramadol can offer a good alternative as it has possible immune enhancing effects and blocks the pro-inflammatory cytokine response [38]. Finally, morphine, fentanyl, oxycodone, and methadone should be avoided due to their immune suppressive effects [39,40]. Morphine for example has an inhibitory effect on IL-2 related to its NF-κB suppression. Fentanyl has different effects on the human immune response.

If anti-inflammatory action is needed in these patients, one should opt for non-steroidal anti-inflammatory drugs (NSAIDs) with anti-viral activity. Based on the recent literature on SARS-CoV-2, there is some emerging evidence for NSAIDs such as indomethacin [40], naproxen [41], flurbiprofen [42], and ibuprofen [42] demonstrating (mostly proven in vitro) anti-viral activity. However, the appropriateness of using antipyretics to treat infectious fever and their effects on the immune system, and clinical outcome remain highly uncertain in this context [40]. Further, there may also be some exciting role of specific NSAIDs that directly target cyclooxygenase-2 (COX-2 inhibitors), an enzyme responsible for inflammation and pain inhibitors, such as celecoxib. Recent experiences in influenza infections showed that the administration of selective COX-2 inhibitors may indeed improve the clinical course of highly pathogenic human invasive viral infections [43]. COX-2 is critical in the inflammatory response process, and deficiency/inhibition of COX-2 results in decreased inflammation and proinflammatory cytokine release, which in turn lead to reduced morbidity and improved survival [44]. 

Previous studies have reported the development of mood disturbances in isolated patients with MPX infection [18]. Mood changes could be a predictable reaction to prolonged isolation without visitors for infection control purposes. The close interface between pain and mood disturbances is of course proven in diverse pathological conditions [45,46,47]. Anxiety or depression requiring counseling was observed in >25% of patients hospitalized with MPX in a 2018 case series from Nigeria [48]. Therefore, health authorities should incorporate the follow-up of patients with MPX infection into a multidisciplinary approach. Treatment with gabapentinoids (such as gabapentin or pregabalin) could be initiated to improve mood disturbances and sleep while reducing pain (certainly in the case of neuropathic pain conditions) [49,50]. Gabapentinoids also exert a strong opioid-sparing effect, but recently, evidence was provided for multi-factorial anti-inflammatory capacities [51,52]. Since patients with MPX infections are obliged to a 21-day home isolation, mental health support should be implemented. Digital mental health intervention may be a feasible and scalable method for addressing mental health issues in this setting. Appropriate digital technologies can be used to combat loneliness and social isolation [53]. However, such systems can also be used to prevent further psychological harm. Previous experiences, for instance in an orthopedic setting of patients with musculoskeletal pain, have shown that such approach reported greater improvements in depression pain interference and physical function of patients than standard care [54]. Patients even reported comparable improvements in depression, anxiety, and pain interference compared with patients who received in-person psychological counseling [54]. In the first instance, a hybrid model of care is recommended, combining innovative digital technologies that are applied for prevention and early intervention strategies in this patient population with more traditional face-to-face approaches. Considering the mandatory isolation, “face-to-face” can be obtained through video consultations safely incorporated into the digital platforms used to monitor patients at home [55].

## 3. Conclusions

The unexpected severity of MPX symptoms has made patient management and their encounters with an already overburdened healthcare system, that was unprepared for this outbreak even more challenging. Continued debilitating pain without proper management for weeks can lay the ground for prolonged nociceptive syndromes, especially in the presence of some neuropathic conditions. Implementation of a comprehensive, holistic approach is promptly needed. Prolonged follow-up should be implemented to quickly diagnose the development of prolonged nociceptive conditions.

In most human infection cases, outpatient management is appropriate and cost-effective, but care must be taken to follow recommended quarantine procedures at home. In addition, intensive daily follow-up with monitoring of vital signs and severity of symptoms should be imposed. Such telemetric follow-up will help patients maintain contact with healthcare workers and receive support and will allow for rapid resolution of symptoms. In addition, mental support should be provided through these systems for a better understanding of stressors and vulnerability as well as for providing faster and better psychological screening and tracking. During the COVID-19 crisis, multiple telemonitoring systems were developed to monitor SARS-CoV-2-infected patients in their home environment [56,57,58]. These systems should be altered to humanize patient care during the current MPX outbreak but can also be very useful for the management of future viral outbreaks. Hence, generic national, and international guidelines on the comprehensive management of pain and other symptoms should be developed and regularly updated.

## Figures and Tables

**Table 1:** Proposed holistic supportive management of acute monkeypox (MPX) infections *.

Component of Management	Symptoms and Clinical Signs	Global Approach	Local/Topical Therapeutic Strategies	Systemic Therapeutic Strategies
Compromised skin (protection)	Skin rash	Do not touch or scratch the rash Avoid secondary infection	Clean with antiseptic Topical benzocaine/lidocaine gels for temporary relief Topical application of Calamine lotion or petroleum jelly to treat itching Apply fucidin/mupironic acid topically Cover with light dressing if extensive lesioning is present	In case of secondary infection, relevant systemic antibiotics should be considered
	Genital (and peri-anal) ulcers	Bathing	Sitz bath Sitting in bathtub with shallow water and adding Epsom salt, vinegar, or baking soda to the water Stool softeners in case of peri-anal ulcers Increase hydration	See section on pain treatment
	Oral ulcers		Warm saline gargles Oral topical anti-inflammatory gel	See section on pain treatment
	Conjunctivitis	Consult ophthalmologist if symptoms persist		
Dehydration	Dizziness, poor appetite, nausea and vomiting, diarrhea	Rehydration and nutritional therapy	ORS or oral fluids Encourage nutritious and adequate diet Intravenous fluids, if indicated	Consider anti-emetics such as ondansetron or promethazine
Symptomatic care	Fever	Exclude surinfection of skin lesions	Tepid sponging	Paracetamol as required
	Headache and/or malaise		Adequate hydration Rest	Paracetamol (short-lasting since medication overuse headache can occur!)
	Dyspepsia			Omeprazole
	Itching/pruritus		Topical calamine lotion Colloidal oatmeal bath	Antihistamine as required (topical treatment options insufficient)
	Pain	Proper identification of type of pain mandatory. Use PQRST pain assessment method to identify pain type. P = Provocation/Palliation Q = Quality R = Radiation/Region S = Severity scale T = Timing scale Use validated questionnaires and clinical sensory examination to distinguish between somatic and neuropathic pain conditions.	Transcutaneous nerve stimulation (TENS) in case of localized pain tDCS (transcranial direct current stimulation) in case of combined pain and depressive symptoms Vagus nerve stimulation in case of pain in cervical region or over different body regions linked to pronounced inflammation	Buprenorphine (Sublingual or transdermal) if required Tapentadol or tramadol as second-line therapy in case buprenorphine fails In case of distinct neuropathic symptoms (screening tool), add gabapentinoids to the analgesic regimen Avoid NSAIDs, but if needed, opt for indomethacin, naproxen, or celecoxib
	Agitation			Diazepam

* Further adapted from the WHO interim rapid response guidance on the clinical management, infection prevention, and control for monkeypox [20]; the CDC guideline on clinical consideration for pain management of Monkeypox [21]; and the guideline by the Government of India for the management of monkeypox disease since it includes several non-pharmacological approaches to symptom management (https://main.mohfw.gov.in/sites/default/files/Guidelines%20for%20Management%20of%20Monkeypox%20Disease.pdf, accessed on 3 July 2022) [25].
